# A pathway to negative acculturation: marital maladjustment mediates the relationship between the length of residency and depressive symptoms in immigrant women in Taiwan

**DOI:** 10.1186/s12905-021-01334-0

**Published:** 2021-05-07

**Authors:** Mei-Huei Lien, Sheng-Shiung Huang, Hao-Jan Yang

**Affiliations:** 1 Department of Public Health, Chung Shan Medical University, No.110, Sec.1, Jianguo N. Rd, Taichung, 40201 Taiwan; 2 Department of Biomedical Engineering, Da-Yeh University, No.168, University Rd., Dacun, Changhua, 51591 Taiwan; 3 Department of Family and Community Medicine, Chung Shan Medical University Hospital, No.110, Sec.1, Jianguo N. Rd., Taichung, Taiwan

**Keywords:** Immigrant women, Acculturation, Marital adjustment, Depressive symptoms

## Abstract

**Background:**

Immigrant women in Taiwan experience a variety of acculturative and marital problems that result in a mental-health problems. We examined the mediational effect of marital adjustment on the relationship between acculturation and depressive symptoms in immigrant women in Taiwan.

**Methods:**

All participants (*N* = 127) were interviewed to collect data regarding their basic sociodemographics, depressive symptoms, acculturation (using language proficiency and years in Taiwan as indicators), and marital adjustment. We used a Sobel test to examine how marital adjustment mediates the relationship between acculturation and depressive symptoms.

**Results:**

Our results indicated that an increased length of residency exacerbated depressive symptoms (β = 0.62, *p* = 0.03) and that this relationship contributed, in part, to the mediational effect of marital adjustment. That is, marital adjustment deteriorated with the length of residency (β =  − 0.26, *p* = 0.0013), resulting in the development of depressive symptoms (β =  − 0.95, *p* = 0.0013).

**Conclusion:**

Although the duration of residency may be useful as a proxy for acculturation in the assessment of some health outcomes, our findings imply that it is better to conceptualize it as a cumulative stress when considering the mental health of immigrant women. Marital maladjustment acts as a mediator in this relationship. As such, it is important to provide immigrant families with programs and resources to assist them in adapting to their marriages and to improve the mental health of immigrant women.

**Supplementary Information:**

The online version contains supplementary material available at 10.1186/s12905-021-01334-0.

## Background

In Taiwan as of March 2020, 561,001 women had immigrated via transnational marriage, comprising 2.4% of the overall population. However, nearly one out of every three transnational marriages ends in divorce, while the proportion for Taiwanese women is about one in four [1]. The higher divorce rate for immigrant women highlights the adjustment problems that exist in transnational marriages.

Marital adjustment refers to the happiness and satisfaction experienced by both partners in a marriage [2] and is significantly correlated with physical and mental health [3]. For the majority of immigrant families in Taiwan, their marriages are typically facilitated by a marriage agency. These rapid transnational marriages are often characterized by the partners not being acquainted with each other prior to the marriage, having a large age difference, speaking different languages, having cultural and background disparities, having a low socioeconomic status, and having different goals for the marriage (mainly, the husband wants to continue his ancestral line and the wife wants to provide her family of origin with financial support) [4]. Lacking an emotional foundation for their relationship, couples in this type of marriage typically show insufficient interaction and lack cohesion, intimacy, and dependability. Some immigrant women experience denigration, criticism, or verbal/physical aggression from their husbands [5]. As a result, adjusting to marriage and family life is more difficult for immigrant women than their Taiwanese counterparts. This is especially the case because women immigrating to Taiwan for marriage move there alone and rarely have social support from outside of their new family. According to the marital-discord model of depression [6], the disadvantages that emerge in transnational marriages may increase the risk of depression in immigrant women, as a result of the increased stress and decreased support they experience.

In general, a long duration of residence in the host country represents successful acculturation, which may benefit the mental health of immigrants [7, 8]. However, in light of the negative acculturation theory [9], which assumes that immigrant health declines with longer residence in the host country due to the loss of protective sociocultural factors and an increase in detrimental lifestyle habits [10], the duration of residence should not always be used as a proxy for acculturation. Rather, it is sometimes better to conceptualize duration of residence as a cumulative stress factor, because it can encompass multiple processes and dimensions of assimilation, only some of which are beneficial for mental health [11, 12]. The declining health advantage over time among the immigrant population has also been supported by several theories. The healthy immigrant theory assumes that immigrants are a highly self-selected population with the best physical and mental health [13, 14]. In contrast, structural theories of immigrant adaptation and health draw attention to the political and economic contexts of immigration, in which resettlement barriers, discrimination, and “othering” may result in immigrants’ health worsening over time [15, 16]. For example, one study showed that immigrant women who had lived in Taiwan for longer had an increased likelihood of marked depression [17]. The explanation proposed by the author was the marked gender roles in Taiwanese society, where immigrant women are confronted with their host country’s cultural norms regarding the motherhood obligations of women. As a result of these norms, immigrant women socializing in public are often met with disapproval from traditional Taiwanese men and their families. The resulting prolonged absence of social interaction increases the risk of depression associated with a longer period of residency in Taiwan.

It is worth noting that Faragallah et al. [18] found that a longer period of residence in the US was also associated with reduced family satisfaction, a measure that is strongly correlated with marital adjustment. This finding implies that the positive relationship between length of residency and depressive symptoms in Taiwan may be mediated by marital maladjustment. This speculation is also partly supported by studies on Mexican immigrant couples, in which the wives’ marital discontent was shown to be uniquely affected by their own depressive symptoms and marital negativity [19]. Moreover, marital satisfaction can be a powerful buffer against various stressful life events [20]. This information clearly suggests that, although the depressive symptoms exhibited by immigrant women are correlated with longer length of stay, this correlation is likely mediated by marital maladjustment. Thus, the purpose of this study was to use a conceptual model to assess the mediating effects of marital adjustment between two acculturation indicators—the length of residency in Taiwan and proficiency in Mandarin—and depressive symptoms in immigrant women.

## Methods

### Participants

The participants in this cross-sectional study were recruited from a population of immigrant women in Central Taiwan using chain referral sampling, which is an appropriate method for research on transcultural issues and hard-to-reach populations [21]. The majority of immigrant women seek the support and assistance of civil immigrant associations for adapting to life in Taiwan, and we selected eligible participants from lists provided by three such associations. These associations have been established mainly to serve immigrant women, especially those who are underprivileged or socioeconomically disadvantaged.

The total number of people served by these three associations is approximately 5000 per year, although our target cases represented only a small proportion of their members. The majority of members were “general” immigrant women who had joined the associations to make friends with people with similar backgrounds and thus establish a social network and support system. As such, enrolling participants from these associations helped to provide sufficient variation to clarify the effects of potential factors. We conducted the sampling at the associations’ premises by selecting eligible immigrant women from among the daily visitors. Eligible participants were marriage immigrant women (a) of Southeast Asian or Chinese origin who were (b) between the ages of 20 and 50.

The participants’ consent to participate in a questionnaire survey was obtained before the association staff gave us the participant lists. Subsequently, accompanied by an association staff member, we traveled to designated locations to interview the participants. We also requested that they nominate other women they deemed eligible for the study. The participants or association staff were instructed to seek initial consent to participate from the nominees by telephone. Once this had been obtained, the referring participant and a trained interviewer from the association accompanied us during the survey process to reduce the new participants’ anxiety and defensiveness. According to their literacy level, the participants underwent either an interview or a questionnaire survey, which required approximately 15–20 min to complete. Each participant was awarded NT$150 for completing the survey and NT$50 for each additional participant they nominated. The interviewers from the immigrant associations were awarded NT$100 for each participant they helped to complete the survey. Ultimately, 127 participants were surveyed. This study was approved by the Chung Shan Medical University Hospital Institutional Review Board (Case no. CS12084).

## Measures

### Depressive symptoms

We used the Center for Epidemiologic Studies Depression Scale (CES-D) to evaluate the depressive symptoms of the participants. This questionnaire has been used in numerous studies and has been proven to possess excellent reliability and validity [22]. The CES-D comprises 20 items, which are scored using a four-point Likert scale. Each item thus had one of four possible answers: *very rarely* (0 points), *rarely* (1 point), *often* (2 points), and *very often* (3 points), resulting in a total score of 0–60 points. A higher score denotes a greater severity of depression. The Cronbach’s α for the CES-D in the present study was 0.88.

### Mandarin proficiency and length of stay in Taiwan

We used Mandarin proficiency and years in Taiwan as two indicators of the acculturation of immigrant women (See Additional file 1: Supplementary material). To assess Mandarin proficiency, a research assistant evaluated listening, speaking, reading, and writing ability during the in-person interview, with each scored according to one of three possible proficiency levels: *fluent* (3 points), *intermediate* (2 points), and *limited* (1 point). Thus, the participants obtained a summed language proficiency score of between 4 and 12 points. For the purposes of analysis, a binary variable was used as in some previous studies [23, 24], such that participants who obtained a score of 4–8 were classified as having low Mandarin proficiency, and those who obtained a score of 9–12 were classified as having high Mandarin proficiency. Cronbach’s α for the acculturation variables proposed in this study was 0.77. The number of years of residency in Taiwan was provided by the participants.

### Marital adjustment

We used a scale for measuring marital adjustment that was proposed by Wu [25]. This scale comprises eight items, which cover facets of marital adjustment such as communication with family members, caring and respect for the partner’s needs and temperament, cooperation in problem solving, and child rearing. Examples of the items are “Do you feel that you have come to a mutual understanding in your marriage since you moved to Taiwan?” and “Do you feel that you get along with all the members of your household after moving to Taiwan?” A four-point Likert scale was used for each item. The participants selected one of four possible answers: *strongly agree* (3 points), *agree* (2 points), *disagree* (1 point), and *strongly disagree* (0 points). Thus, the participants obtained an adjustment score of between 0 and 24 points. A high score denotes highly favorable marital adjustment conditions. Cronbach’s α for this marital adjustment scale was 0.82.

### Control variables

Age, marital status, the education level of the husband and wife, personal income, occupation, and the woman’s original nationality were used as the control variables. The women were categorized into two groups according to their marital status: married and divorced/widowed.

### Data analysis

We analyzed the data using SAS 9.4 software. A descriptive analysis was used to assess the distribution of the sociodemographic variables. We then calculated Pearson’s or Spearman’s correlation coefficients to provide an understanding of the correlation between and directionality of the three control variables (language proficiency, years in Taiwan, and marital adjustment) and the participants’ depressive symptoms. Finally, we used a Sobel test to examine how marital adjustment mediates the relationship between language proficiency and years in Taiwan and depressive symptoms. To conduct the Sobel test, direct effects (represented as *c*′) were subtracted from the total effects (represented as *c*) between the predictive variable (language proficiency and years in Taiwan) and the outcome variable (depressive symptoms). A difference between *c* and *c*′ that is significantly different from zero indicates the existence of mediational effects. The 95% confidence interval (CI) for the difference was calculated using a bootstrapping method. The number of simulations was set to 5000 to ensure that the 95% CI had converged and was stable. To obtain regression coefficients and their standard errors (SE) for the Sobel test, we performed multiple regression analyses controlling for age, personal income, husband’s income, education level, husband’s education level, employment status, and original nationality. Under this analytical framework, with a significance level of 0.05, a statistical power of 0.8, and a medium effect size of 0.5, the required sample size was determined to be *n* = 81 [26].

## Results

A total of 127 immigrant women completed the questionnaire; 18 were divorcees (14%), 12 were widows (9%), and 97 were married (76%). Approximately half of the participants had resided in Taiwan for 10–14 years (48%) and were currently raising two children (46%), and approximately two-thirds (65%) were between the ages of 30 and 39. Most of the participants (89%) and their spouses (79%) had an educational level of high school or lower. A substantial proportion of the participants (57%) and their spouses (38%) earned a monthly salary of NT$15,000–29,999. These statistics suggest that the socioeconomic status of the participants was generally low, and only 43% of the participants demonstrated favorable proficiency in Mandarin (Table 1).

**Table 1:** Sociodemographic distribution and mean scores for depressive symptoms (measured using the CES-D), stratified by the sociodemographic variables, of the immigrant women (N = 127)

Variables	*n*	%	Mean	SD
Marital status^a^				
Married	97	76.38	12.66	7.72
Divorced	18	14.17	23.56	14.70
Widowed	12	9.45	27.33	10.86
Number of children				
0	18	14.17	15.11	11.67
1	41	32.28	14.73	10.46
2	59	46.46	16.78	10.86
3+	9	7.09	13.90	8.75
Age				
25–29	17	13.39	13.76	10.14
30–34	48	37.80	13.75	8.95
35–39	34	26.77	17.18	11.47
40+	28	22.05	18.32	12.08
Education				
Primary school or lower	34	26.77	14.91	10.39
Middle school	39	30.71	16.46	10.80
High school	40	31.50	16.20	11.53
College/university or above	14	11.02	13.86	8.47
Education of husband				
Primary school or lower	24	18.90	20.17	12.78
Middle school	33	25.98	16.00	8.95
High school	46	36.22	14.39	10.48
College/university or above	24	18.90	13.21	9.86
Personal monthly income (NTD)				
0	16	12.60	15.69	12.10
1–14,999	22	17.32	16.73	8.18
15,000–29,999	72	56.69	15.86	10.99
30,000–49,999	13	10.24	14.85	11.59
≥ 50,000	4	3.15	9.25	8.62
Husband’s monthly income (NTD)				
0	16	12.60	22.06	11.21
1–14,999	5	3.94	24.40	16.27
15,000–29,999	49	38.58	14.71	9.23
30,000–49,999	45	35.43	14.40	10.65
≥ 50,000	12	9.45	12.25	9.02
Job of immigrant women				
Public servant	0	0.00	–	–
Businesswoman	16	12.60	12.81	9.70
Agriculture	7	5.51	12.71	7.95
Labor	43	33.86	16.56	10.34
Service industry	35	27.56	16.17	11.85
Unemployed	16	12.60	16.81	11.75
Others^b^	10	7.87	15.00	9.58
Job of husbands				
Public servant	2	1.57	18.50	6.36
Businessman	17	13.39	12.35	10.54
Agriculture	11	8.66	12.18	7.49
Labor	52	40.94	15.73	9.63
Service industry	16	12.60	14.94	12.10
Unemployed	12	9.45	22.83	13.08
Others^c^	17	13.39	16.50	11.70
Employment status				
Full time	93	73.23	15.51	10.17
Part time	16	12.60	17.44	12.56
Unemployed	18	14.17	15.81	12.04
Employment status of husband				
Full time	106	83.46	14.74	9.99
Part time	7	5.51	18.86	12.88
Unemployed	14	11.02	22.75	13.27
Original nationality				
Vietnam	93	73.23	15.98	10.61
Thailand	1	0.79	27.00	–
Philippines	2	1.57	19.00	14.14
Mainland China	25	19.69	14.08	11.41
Indonesia	6	4.72	14.67	7.79
City of residence				
Taichung	49	38.58	14.96	11.67
Changhua	75	59.06	15.37	9.18
Nantou	3	2.36	35.00	12.17
Mandarin language proficiency				
Low	73	57.48	17.01	11.25
High	54	42.52	13.87	9.51
Years in Taiwan				
0–4	15	11.81	10.80	6.62
5–9	43	33.86	14.72	9.35
10–14	61	48.03	16.80	11.43
15–20	8	6.30	21.38	13.93

*CES-D* center for epidemiologic studies-depression scale, *SD* standard deviation, *NTD* new Taiwan dollar (1 NTD = 0.033 USD at the time of the study)

^a^Significantly associated with the CES-D score, as indicated by the generalized linear model, after the other variables in this table had been controlled for

^b^Including home OEM, translator, and international trade secretary

^c^Including mason, civil engineer, plumber, and electrician

The mean score on the CES-D for the total sample was 15.68 (SD: 10.62). Despite the CES-D scores varying widely with some of the sociodemographic variables, the generalized linear model showed that only marital status had a significant effect on the CES-D score when the other variables in Table 1 were controlled for. In other words, divorced or widowed immigrant women had more depressive symptoms than those who were married.

In the correlation matrix (Table 2), Mandarin proficiency was not significantly correlated with the other variables. A longer residency in Taiwan was correlated with a higher risk of depressive symptoms (*r* = 0.24, *p* = 0.0076) and poorer marital adjustment (*r* =  − 0.29, *p* = 0.0009). Marital adjustment was also correlated with depressive symptoms. The participants who demonstrated good marital adjustment showed fewer depressive symptoms (*r* =  − 0.34, *p* = 0.0001).

**Table 2:** Correlation coefficients between study variables

Variable	1	2	3	4	5	6	7	8
1. Depressive symptoms	–							
2. Mandarin language proficiency	− 0.12	–						
3. Years in Taiwan	0.24**	− 0.02	–					
4. Marital adjustment	− 0.34***	0.16	− 0.29***	–				
5. Marital status	0.49***	0.01	0.32***	− 0.41***	–			
6. Women’s income	− 0.07	0.04	0.16	0.08	0.08	–		
7. Education	− 0.01	0.22*	− 0.03	0.08	0.06	− 0.02	–	
8. Husband’s income	− 0.26**	0.16	− 0.11	0.09	− 0.18*	0.14	0.17	–
9. Husband’s education	− 0.24**	0.25**	− 0.08	0.26**	− 0.23*	0.10	0.13	0.34***

Coefficients were calculated with Pearson correlation method or Spearman correlation method where necessary

^*^p < 0.05, **p < 0.01, ***p < 0.001

These results suggest that duration of residency in Taiwan influences marital adjustment, leading to the occurrence of depressive symptoms. We therefore used a series of multiple regression analyses to test the total and direct effects of length of residency on depressive symptoms (Table 3). Mandarin proficiency was not included in the model because it was not associated with depressive symptoms in the correlation analyses. We also assessed the indirect effect of acculturation through marital adjustment. After controlling for sociodemographic and socioeconomic variables, we found that the duration of residency in Taiwan had a significant effect on depressive symptoms (β = 0.62, *p* = 0.0225): the longer the residency in Taiwan, the worse the depressive symptoms. However, when the indirect effect of marital adjustment was taken into account, the direct effect of duration of residency in Taiwan became nonsignificant (β = 0.40, *p* = 0.1410). It is noteworthy that marital adjustment was negatively associated with depressive symptoms in both the direct effects model (β =  − 0.82, *p* = 0.0072) and the indirect effects model (β =  − 0.95, *p* = 0.0013), suggesting that it has a mediational effect.

**Table 3:** Total, direct, and indirect (mediated by marital adjustment) effect models of years in Taiwan on depressive symptoms

Variables	Total effect model			Direct effect model			Indirect effect model		
	β	s.e. (β)	*p*	β	s.e. (β)	*p*	β	s.e. (β)	*p*
Years in Taiwan [predictive variable]	0.62*	0.27	0.0225	0.40	0.28	0.1410	–	–	–
Marital adjustment [mediator]	–	–	–	− 0.82*	0.30	0.0072	− 0.95*	0.29	0.0013
Constant and controlled variables									
Constant	15.50	8.40	0.0675	31.22*	10.00	0.0023	33.55*	9.92	0.0010
Age (years)	− 0.02	0.19	0.9302	− 0.02	0.18	0.9322	0.08	0.17	0.6241
Women’s income (NTD)	− 0.56	1.25	0.6236	− 0.61	1.21	0.6164	− 0.37	1.21	0.7582
Husband’s income (NTD)	− 1.70	0.91	0.0641	− 1.88*	0.89	0.0370	− 1.94*	0.89	0.0314
Education (years)	0.90	1.06	0.3973	1.12	1.04	0.2829	1.16	1.04	0.2699
Husband’s education (years)	− 1.69*	0.84	0.0476	− 1.12	0.84	0.1874	− 1.01	0.85	0.2343
Employment status (Ref. = full time)	0.36	0.89	0.6850	− 0.17	0.89	0.8454	− 0.23	0.90	0.7938
Original nationality (Ref. = Vietnam)	0.06	0.46	0.8990	0.08	0.45	0.8640	− 0.05	0.44	0.9067

^*^p < 0.05

**Fig. 1:**
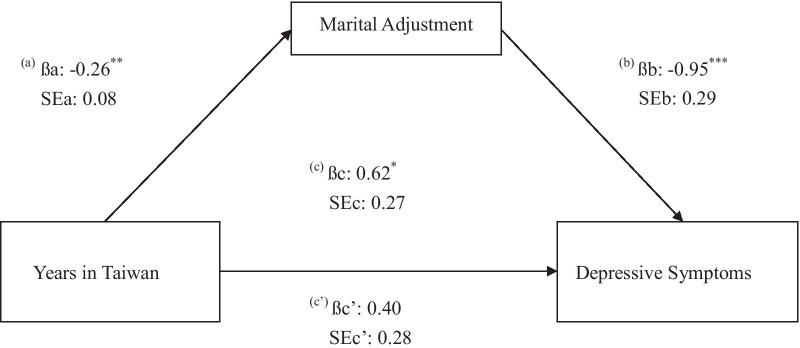
Mediational effect of marital adjustment between years in Taiwan and depressive symptoms among immigrant women by using Sobel test. Path (a) modeled marital adjustment as a dependent variable and years in Taiwan as an independent variable (ßa and SEa are the regression coefficient and its standard error, respectively in the model); Path (b) modeled depressive symptoms as a dependent variable and marital adjustment as an independent variable (ßb and SEb are the regression coefficient and its standard error, respectively in the model); Path (c) modeled depressive symptoms as a dependent variable and years in Taiwan as an independent variable (ßc and SEc are the regression coefficient and its standard error, respectively in the model); Path (c’) is the model c added in the marital adjustment as a control variable (ßc’ and SEc’ are the regression coefficient and its standard error, respectively in the model). All models have controlled for age, personal income, husband’s income, education, husband’s education, employment status, and original nationality. *p < 0.05, **p < 0.01, ***p < 0.001

Based on these results, we conducted the Sobel test to analyze the mediational effects (Fig. 1). After controlling for potential confounding factors, we found that participants who had lived in Taiwan for longer had poorer marital adjustment (βa =  − 0.26, SE = 0.08, *p* = 0.002), which resulted in higher depressive-symptom scores (βb =  − 0.95, SE = 0.29, *p* = 0.0013). Based on these data, the Sobel test statistic was 2.31, with a corresponding *p* value of 0.0210, indicating that marital maladjustment mediated the relationship between length of residency and poor mental health.

## Discussion

The present study shows that the depressive symptoms of immigrant women living in Taiwan are associated with their duration of residency and marital adjustment. In contrast to previous research, this study shows that the longer the residency, the worse the depressive symptoms become. However, this positive relationship between the length of residency and depressive symptoms may be an epiphenomenon mediated by marital maladjustment.

Although the duration of residence has been considered a reasonable surrogate measure for acculturation in some previous studies [7, 8], our results suggest that a longer stay in Taiwan hampers the mental health of immigrant women. That is, it may be better to conceptualize longer periods of residency in the host country as a cumulative stress, rather than as a sign of acculturation, when considering mental health in immigrant women. Our findings are in agreement with the phenomenon described by negative acculturation theory, which posits that with longer residence in the host country (originally the US), poor health or health behaviors can be driven by unhealthy cultural changes as the local lifestyle is adopted [9]. Nevertheless, much of the research thus far that has examined the negative acculturation theory has focused on chronic and physical outcomes, such as cardiovascular diseases and metabolic-syndrome–related disorders [27, 28]. Relatively few studies have verified whether psychological outcomes also follow the negative acculturation effect. In addition, the length of residence may not fully capture the acculturation process, which consists of a variety of complex mechanisms and is affected by both personal- and social-level factors. For example, the greater the proportion of their lives spent in the US, the poorer the health and health behaviors of Korean immigrants with less education, whereas those with more education tended to exhibit more healthy behaviors [29]. This highlights the moderating effect of education on health when measuring acculturation by length of residency. This is partly supported by our findings from the regression models, where the husband’s income and education level were negatively associated with their wives’ depressive symptoms. Similarly, the relationship between length of residency and health can also be moderated by ethnicity, age, gender, family, and community reception [12, 30]. The exact mechanisms and pathways to health that are encompassed within the duration of residency are subject to interpretation [31]. Further, since the mechanisms determining mental health are, in general, much more complicated than those determining physical health, the relationship between the length of residence and depression may operate via several alternative pathways.

We found that the relationship between duration of residence in Taiwan and depressive symptoms was mediated by marital adjustment: participants who had lived in Taiwan for longer exhibited poorer marital maladjustment. Thus, poor marital adjustment exacerbates depressive symptoms. After taking marital adjustment into account, we found that the effect of duration of residence in Taiwan on depressive symptoms became nonsignificant. This reveals a fully mediational effect [32] of marital maladjustment and that the relationship between the length of residence and depressive symptoms is an epiphenomenon. In immigrant households in Taiwan, marital-adjustment problems often develop gradually, due to a lack of affection in the marriage and a poor relationship between the wife and her mother-in-law [33–36]. Most couples in immigrant families do not possess an emotional foundation to their relationship [37]. Instead, they are brought together rapidly by marriage agencies to satisfy the distinct goals of the marriage partners (e.g., the husband’s desire to continue his family line and the wife’s desire to improve the financial position of her family in her country of origin). In such households, the tolerance and understanding between the couple gradually deteriorates after they have achieved their separate goals, and marital satisfaction slowly decreases. This may explain why a longer residency in Taiwan is negatively correlated with marital adjustment. Furthermore, approximately 70% of immigrant families in Taiwan live with extended family [14], in other words the immigrant women live not only with their husband and children, but also with other relatives of their husband. This is a traditional living arrangement in Asian society. Conflict with anyone in the family is likely to damage the marriage relationship. As such, marital adjustment, to some extent, refers to the ability to get along with all the members of the household, which is similar to family-relationship adjustment in other cultures. For example, in a family in which three generations live together, the immigrant women are required not only to resolve problems between them and their husbands, but also to resolve those with their mothers-in-law and children, which might also indirectly result in poor marital adjustment. The problems that such intergenerational cultural differences generate are not easily resolved over time [38]. These problems cause marital adjustment to deteriorate over time, resulting in depression.

This study offers insight into the negative effects of a long residency in Taiwan on depressive symptoms in immigrant women. Specifically, we identified a potential mechanism via mediation analyses guided by theoretical frameworks, and found that marital maladjustment mediates the relationship between length of residence and depressive symptoms. Women who immigrate via transnational marriage are a hard-to-reach population, but these preliminary findings may provide a helpful direction for future research. However, several limitations should be taken into account when considering the results presented in the current study. First, acculturation is a multidimensional concept, and the assessment of acculturation used in this study (using language proficiency and duration of residency in Taiwan) may not be robust or sound [39]. Second, this is a cross-sectional study and is thus unable to elucidate the time course of the causal relationships between the variables. It is also possible that the immigrant women first become depressed, and that this then affects their marriage. Third, the diverse and limited sample examined in this study may limit its statistical power and produce unstable estimates. For example, we included divorced and widowed women in the analysis because we also wanted to investigate whether marital status contributed to the mental health of immigrant women. However, we had no choice but to ask these women to complete the marital adjustment scale with reference to their former husband, which may have introduced recall bias into the study and confused temporality between the variables. In addition, women who were suffering from depressive symptoms or maladjusted in their marriage may not have been willing or able to participate in the study. This may have resulted in a nonparticipant bias. Fourth, because we used a chain referral sampling method, the majority of the women included in the study were from Vietnam. This bias toward one ethnic group may have created problems related to representation and generalization.

## Conclusion

In conclusion, length of residency had a negative (i.e., exacerbating) effect on depressive symptoms in immigrant women in Taiwan. However, this negative acculturation effect on mental health appears to be an epiphenomenon that may occur via an alternative pathway resulting from the mediating effect of marital maladjustment. Couples in transnational marriages often experience greater difficulty in overcoming cultural, social, and familial challenges than those in marriages in general because the emotional foundation of their relationship is weaker. In this context, the marital adjustment and satisfaction of immigrant women decrease the longer they stay in Taiwan, directly and indirectly causing depressive symptoms. Our findings suggest that programs or resources should be provided to assist immigrant families to adapt to married life and to reduce depression in the women.

## Supplementary Information

**Additional file 1.** Acculturation of cross-cultural immigrant women in Taiwan.

## Data Availability

The datasets used and analyzed during the current study are available from the corresponding author on reasonable request.

## Abbreviations

CES-D: Center for epidemiologic studies-depression scale
SD: Standard deviation
SE: Standard error
