# Supplementary material for: A pathway to negative acculturation: marital maladjustment mediates the relationship between the length of residency and depressive symptoms in immigrant women in Taiwan
Source: BMC Womens Health. 2021 May 7;21:190. doi: 10.1186/s12905-021-01334-0 (PMC8103576; doi:10.1186/s12905-021-01334-0)
Supplement: Supplementary file 1 — Additional file 1. Acculturation of cross-cultural immigrant women in Taiwan. [file 12905_2021_1334_MOESM1_ESM.docx]

Acculturation of cross-cultural immigrant women in Taiwan

1. Language proficiency

| Language type | Listening | Speaking | Reading | Writing |
| --- | --- | --- | --- | --- |
| Mandarin | □ fluent  □ intermediate  □ limited | □ fluent  □ intermediate  □ limited | □ fluent  □ intermediate  □ limited | □ fluent  □ intermediate  □ limited |
| Hokkien | □ fluent  □ intermediate  □ limited | □ fluent  □ intermediate  □ limited | □ fluent  □ intermediate  □ limited | □ fluent  □ intermediate  □ limited |
| Hakka | □ fluent  □ intermediate  □ limited | □ fluent  □ intermediate  □ limited | □ fluent  □ intermediate  □ limited | □ fluent  □ intermediate  □ limited |

1. Years in Taiwan

How many years have you been in Taiwan? _________

Arrived at Taiwan on ________(yyyy)_____(mm)_____(dd)
